# Measurement of the Silver Ion Concentration in Wound Fluids after Implantation of Silver-Coated Megaprostheses: Correlation with the Clinical Outcome

**DOI:** 10.1155/2013/763096

**Published:** 2013-05-29

**Authors:** B. Hussmann, I. Johann, M. D. Kauther, S. Landgraeber, Marcus Jäger, S. Lendemans

**Affiliations:** ^1^Trauma Surgery Department, University Hospital Essen, Hufelandstraße 55, 45122 Essen, Germany; ^2^Orthopaedic Department, University of Duisburg-Essen, Hufelandstraße 55, 45122 Essen, Germany

## Abstract

*Background.* Tumor patients and patients after traumas are endangered by a reduced immune defense, and a silver coating on their megaprostheses may reduce their risks of infection. The aim of this study was to determine the silver ion concentration directly measured from the periprosthetic tissue and the influence on the clinical outcome. * Material and Methods.* Silver ions were evaluated in 5 mL wound fluids two days postoperatively and in blood patients 7 and 14 days after surgery using inductively coupled plasma emission spectrometry in 18 patients who underwent total joint replacement with a silver-coated megaendoprosthesis. * Results.* The concentration of silver ions averaged 0.08 parts per million. Patients who showed an increased silver concentration in the blood postoperatively presented a lower silver concentration in the wound fluids and a delayed decrease in C-reactive protein levels. There were significantly fewer reinfections and shorter hospitalization in comparison with a group that did not receive a silver-coated megaprosthesis. * Conclusion.* An increased concentration of silver in the immediate surroundings of silver-coated prostheses was demonstrated for the first time in cohorts of patients with trauma or tumors. An elevated concentration of silver ions in the direct periprosthetic tissue may have reduced the infection rate.

## 1. Introduction

Applying megaprostheses to reconstruct osseous defects after trauma, tumor, or infection has been well established for decades. These implants can replace critical size osseous defects in long bones, such as in total joint revisions or after local tumor resections [1–3]. One therapeutic challenge is the high infection rate in the latter group, which is approximately 35% in these patients compared to 1-2% in healthy individuals. Therefore, some authors advocate using silver-coated prostheses in this special cohort [4]. Silver ions have a bactericide effect because they can attach to the DNA and thus inhibit protein synthesis [4, 5]. Moreover, it is evident that silver ions induce resistance to bacteria [6, 7].

Currently, silver-coated prostheses are applied mainly due to the following two indications: (1) for infection prophylaxis in tumor endoprosthetics and (2) as the last option for patients after extensive trauma-related infection.

*In vitro* studies have demonstrated the efficiency of silver ions compared to other metals in killing *Staphylococcus epidermidis* [8]. In rabbits, Gosheger et al. showed reduced infection rates after the implantation of silver-coated prostheses [9]. In a recent clinical trial, Hardes et al. presented a reduction of the infection rate to 5.9% in tumor patients compared to 17.6% in a control group [10]. However, there is still a lack in clinical data, and the current literature almost exclusively includes patient cohorts after tumor surgery. Adequate large studies for total joint revision surgeries with silver-coated endoprostheses do not yet exist.

At this time, the typical toxic side effects of silver, such as dermal argyria (i.e., blue or bluish-grey colored skin), ocular argyrosis, gastroenteritis or fever, have not been associated with silver-coated endoprostheses. Neither Gosheger et al. nor Hardes et al. could determine evident toxicological side effects of silver in an animal experiment and in a prospective clinical study, respectively [3, 9]. This corresponds to Jung et al., who found only a mild toxicity of silver ions to human cells [11]. Recent data in the literature have demonstrated a systemic accumulation of silver ions in blood and urine as well as in local tissues adjacent to the silver-coated implant [3]. To the best of our knowledge, current studies regarding the silver concentrations in the immediate surroundings of prostheses do not exist so far.

Thus, we initiated a prospective clinical study to determine the silver ion concentration after the implantation of silver-coated prostheses that appeared in the wound fluids extracted from the immediate surroundings of the prosthesis. Questions addressed included the following.

1. Can the silver ion concentration be directly measured from the periprosthetic tissue using wound fluids?
2. Is this silver ion concentration toxic for the patient?
3. Does the use of silver-coated prostheses have an influence on the clinical course or outcome of the patient?

## 2. Material and Methods

Patients were examined with the following inclusion criteria.

1. Age ≥ 18 years.
2. An indication for the implantation of a silver-coated endoprosthesis, either due to revision surgery after trauma or due to infection prophylaxis in oncologic patients with a malignant process of the bone.
3. The informed consent of the patients to conduct the study.

Exclusion criteria were as follows.

1. The refusal of the patient to conduct the study.
2. The existence of other silver-coated implants (e.g., silver-coated stents) in the patient.
3. A known allergy or hypersensitivity reaction against silver in the previous medical history of the patient.

After the full approval by the local ethical committee, patients were recruited prospectively between 2008 and 2012. All patients underwent surgery in the Traumatology Department of the University Hospital Essen. Only modular silver-coated prostheses from the company Implantcast were implanted (Implantcast Co., Buxtehude, Germany) and fixed with polymethyl methacrylate (PMMA) bone cement. The silver coatings of the titanium-vanadium megaprostheses were realized by a galvanic deposition of elementary silver (percentage purity of 99.7%) on the prosthetic surface. The layer thickness ranged from 10 to 15 mm. Additionally, a 0.2 mm-thick gold layer between the titanium-vanadium surface of the prosthesis and the silver-coating was necessary to enable the sustained release of silver ions in the periprosthetic tissue and prevent progressive corrosion. No silver coating was applied at the articulating surfaces or the prosthetic stems [3].

### 2.1. Patients

Eighteen patients fulfilled the inclusion criteria and were available for evaluation; 11 of them were female. The average age at the time of surgery was 60.1 years (SD 19.4 years). The indication for the implantation of a silver-coated endoprosthesis was due to an infection for 10 patients, and 8 patients received a silver-coated primary endoprosthesis or a prosthesis replacement in the case of aseptic loosening of the primary nonsilver-coated prosthesis due to an infection prophylaxis from a tumor (Table 1). As shown in Table 1, two of the cases after trauma were secondary open fractures. In addition, Table 1 presents the corresponding total silver mass of the applied silver prostheses in grams.

In the infection group, an infection was noted, on average, 18 days postoperatively.  Table 2 demonstrates the pathogens detected using microbiological analysis. In two cases, no pathogens from the intraoperative smears and tissue samples could be proven by either clinical or laboratory findings. Due to the small sample size of the infection group, it was not possible to demonstrate correlation between the duration of the surgical procedure, age, tumour disease, and/or trauma severity or blood loss and the infection rate. In the current literature, this relation has been clearly described by Ahrens et al. [4]. In the infection group, the infected osteosynthesis or total endoprosthesis was removed, and a temporary antibiotic-impregnated cement spacer was implanted for 6 weeks before the implantation of the silver-coated megaprosthesis. Furthermore, patients received a 6-week pathogen-specific antibiotic treatment (intravenously or orally) prior to the implantation of the silver-coated megaprosthesis. In each patient, a trial removal of the infected tissue was performed previously, and the megaprosthesis was implanted only if there were no pathogens in the tissue samples. On the day of the implantation of the silver-coated megaprosthesis, all patients—those in the infection group and those in the infection prophylaxis group—received three doses of perioperative intravenous antibiotic prophylaxis with a cephalosporin over 24 hours. No other antibiotics were administered.

### 2.2. Sample Collection

**Figure 1:**
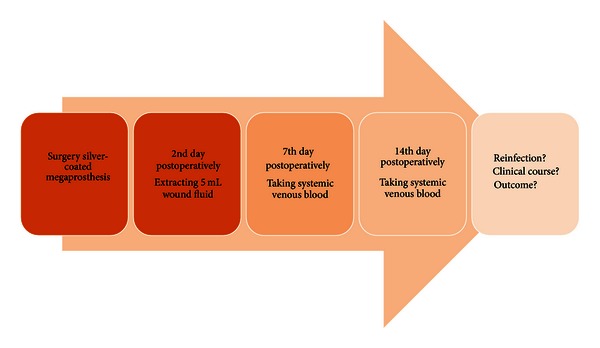
Graphic representation of test procedure.

Two days after surgery, blood samples were collected using the Redon drains that had been placed immediately surrounding the implant. The Redon bottles were shaken; directly afterwards, exactly 5 mL was extracted for analysis and preserved in serum Monovettes (S-Monovettes, Co., Sarstedt, Nümbrecht, Germany). In the case of more than one Redon bottle being used, the fluid of each Redon bottle was analyzed individually, and a total mean value was established for each evaluated patient. The measurement of the silver ion concentration—at the time when samples were collected from the Redon bottle—was the main focus, irrespective of the amount of wound secretion. The total amount of silver ions released from the prosthesis was not part of the evaluation. There were only minor differences between the groups with regard to the amount of wound secretion on the second postoperative day. In the same way, exactly 5 mL of systemic venous blood from each patient was sampled on the 7th and 14th day postoperatively. During this time, no patient in the study underwent additional surgery (Figure 1). 

### 2.3. Sample Preparation and Analytics

#### 2.3.1. Sample Preparation

The whole blood or serum samples were heated at 300°C and denatured in 65% nitric acid (Merck Co., Darmstadt, Germany) and 35% hydrogen peroxide (Merck Co., Darmstadt, Germany). The clear fluid resulting from this was subsequently preserved for analysis in Falcon tubes at −80°C (Greiner bio-one, Heidelberg, Germany).

#### 2.3.2. Analytics

Based on the established technique described by Rahil-Khazen et al. to verify metal ions from human tissue samples, the analysis was performed using mass spectrometrically and inductively coupled atomic absorption spectrometry (ICP-AAS) [12]. The samples were exclusively analyzed for silver ions. To receive statistically reliable results, each probe was measured in triplicate. The detection limit for silver ions of this analytic procedure was 0.01 parts per million (ppm).

### 2.4. Classification

The collective group was analyzed, and two group-specific analyses were carried out as follows: (1) silver-coated endoprosthesis due to an infection versus infection prophylaxis; (2) subgroup analysis depending on the mean value of the silver ions in wound fluids (MV 0.08 ppm) (high silver group: >0.08 ppm, low silver group: <0.08 ppm). Over the same period of time, 6 patients were implanted with a nonsilver-coated endoprosthesis. These patients formed the control group for detecting naturally occurring silver in the blood to determine the standard value. 

**Figure 2:**
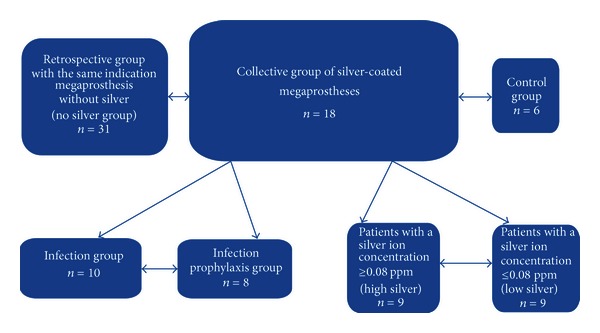
Group distribution and classification of the subgroups.

Furthermore, a retrospective comparative analysis regarding the infection prophylaxis from local case material (group: no silver) in patients without silver-coated endoprosthesis implantation (*n* = 31 patients) was carried out from 2004 to 2011 (Table 3). The classification is shown in Figure 2.

### 2.5. Clinical Course and Outcome

The patients were examined based on the following criteria.

-  Laboratory determination of C-reactive protein (CRP) (high correlation with the course of a bacterial infection) [13].
-  Reinfections and concomitant revision surgery (until 12 months postoperatively).
-  Duration of stay in the hospital.
-  Postoperative function (until 12 months postoperatively).
-  Survival rate of the implant (until 12 months postoperatively)

### 2.6. Statistics

The statistical analysis was carried out with the Statistic Package for Social Science (SPSS, version 19, IBM, Chicago, IL, USA). The data were analyzed for significance by the Mann-Whitney *U* test and the Wilcoxon signed-rank test. Differences between the groups were evaluated using a *t*-test for continuous variables, and correlation tests were carried out according to Pearson (*c * = correlation). In total, only a restricted statistical statement can be made due to the small group sizes. A *P* value of <0.05 was considered significant.

## 3. Results

### 3.1. Mass Spectrometry (ICP-AAS) of the Wound Fluid

**Figure 3:**
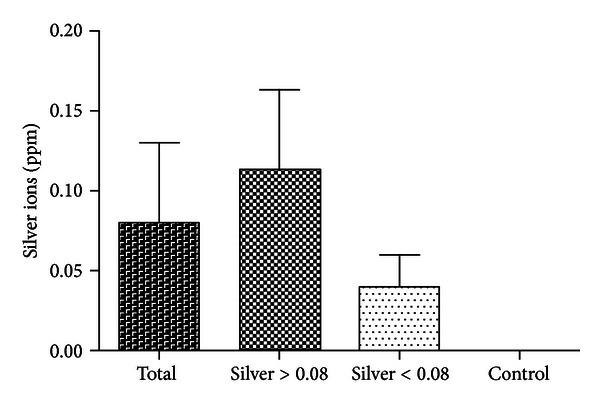
Measurement of silver ions in wound fluids (mean value). ppm: parts per million.

In all 18 patients, increased silver ions could be detected in the samples from the wound fluids of the Redon bottles. The mean value of this silver ion concentration was 0.08 ppm (SD 0.05). There was no significant difference between the silver ion concentration in the infection group (MV: 0.07 ppm, SD 0.1) and the infection prophylaxis group (MV: 0.08 ppm; SD 0.14; *P* ≥ 0.05) (Figure 3). Additionally, no correlation between the silver ion concentration and the total silver mass of the implanted endoprosthesis could be determined (*c* = 0.2; *P* ≥ 0.05). 

### 3.2. Mass Spectrometry (ICP-AAS) of the Venous Systemic Blood

#### 3.2.1. 7*th* Day Postoperatively

**Figure 4:**
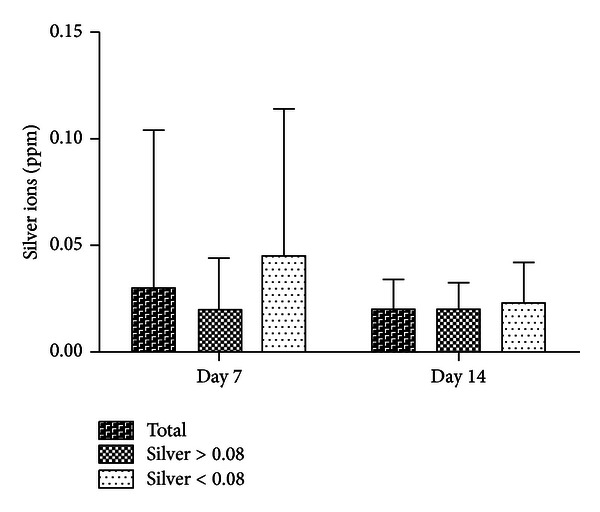
Measurement of silver ions in the venous systemic blood 7 and 14 days postoperatively. Subdivision into two groups depending on the measured mean value of the silver concentration in the wound fluids (mean value: 0.08 ppm). CRP: C-reactive protein; 7th and 14th day postoperatively; ppm: parts per million.

In 7 patients, relevant amounts of silver ions were detected from venous blood. The mean value of the concentration was 0.03 ppm. Four patients with systemic proof of silver ions were members of the infection group (silver ion concentration MV: 0.02 ppm, SD: 0.02). In three patients from the infection prophylaxis group, the mean value of the concentration was 0.05 ppm (SD: 0.06). No significant difference between the groups was demonstrated. Moreover, there was no correlation between the silver ions in the wound fluids and the silver concentration in the systemic blood seven days postoperatively (*c* = 0.22; *P* ≥ 0.05). There was almost no statistical correlation between the total silver mass of the endoprosthesis and the silver concentration in the blood (Figure 4).

#### 3.2.2. 14 Days Postoperatively

Relevant silver concentrations were noted in 11 patients (MV: 0.02 ppm, SD: 0.01). Seven of these patients belonged to the infection group; thus, this group was again the best represented (silver concentration MV: 0.02 ppm, SD: 0.01). As in the preceding sections, no significant differences among the groups could be determined. There was a correlation between the measured silver ions in the venous blood of the collective group and the silver mass of the prostheses (*c* = 0.6; *P* = 0.03). Additionally, there was a significant difference in the silver ion concentration of the venous blood compared to the control group (control group: MV 0; *P* = 0.03). In the subgroup analysis, the silver ions in the wound fluids increased on the 7th day postoperatively in the low-silver group compared to the high-silver group and decreased to the same level on the 14th day (Figure 4). In the control group, silver ion concentrations were discovered neither on the 7th nor 14th days postoperatively. 

### 3.3. CRP

**Figure 5:**
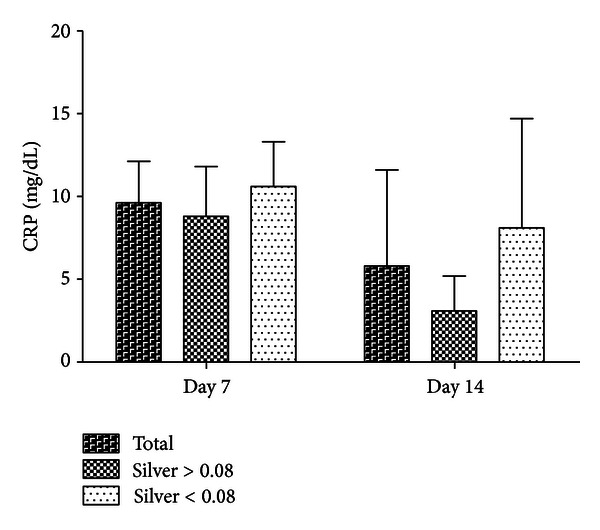
Course of the C-reactive protein in the subgroup comparison depending on the measured mean value of the silver concentration in the wound fluids (mean value: 0.08 ppm) on the 7th or rather the 14th day postoperatively. CRP: C-reactive protein.

After seven days, the CRP mean value in the collective group was 9.6 mg/dL, and after 14 days postoperatively, it was 5.8 mg/dL (normal value < 0.05 mg/dL). Regarding the subgroups, the CRP decreased faster 7 days postoperatively in the high-silver group (*n* = 9; 5 of them were from the infection group) compared to the low-silver group (*n* = 9; 4 of them were from the infection group). Corresponding results were detected 14 days after surgery. After 14 days, the low-silver group reached a similar CRP value as that of the high-silver group on postoperative day 7 (Figure 5).

When analyzing the infection group compared to the infection prophylaxis group, there was no significant difference in the kinetics of the CRP. A correlation between the presence of silver ions in the wound fluids or in the systemic blood and the course of the CRP could not be proven (*c* = 0.28; *P* = 0.34).

### 3.4. Clinical Course and Outcome

#### 3.4.1. Reinfection Rate

Of the 18 examined patients, one patient from the infection prophylaxis group showed a reinfection (5.6%) with *Enterococcus faecalis* three months postoperatively. In this case, a femoral amputation was carried out. This patient had a silver ion concentration in the wound fluid that averaged 0.03 ppm and belonged to the group with less than 0.08 ppm silver ions in the wound fluid (low-silver group). In the infection group, no reinfections were detected within 12 months postoperatively.

**Figure 6:**
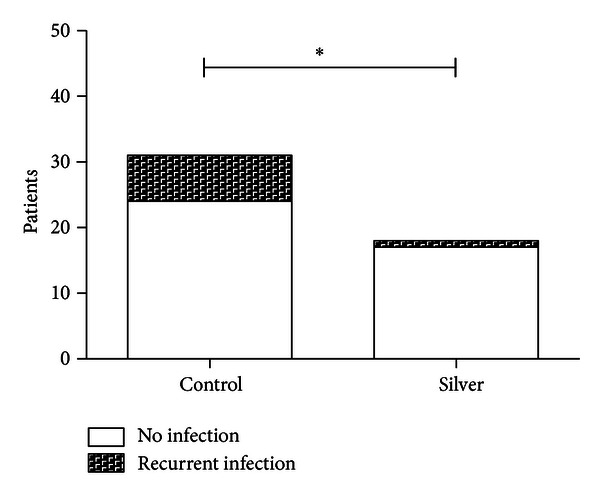
Rate of reinfections in comparison to the no silver group. *P ≤ 0.05.

In comparison to that result, 7 patients in the retrospective group with megaprosthesis without silver showed a significant reinfection rate (22%, *P* = 0.01) (Figure 6).

#### 3.4.2. Duration of Stay in the Hospital

**Figure 7:**
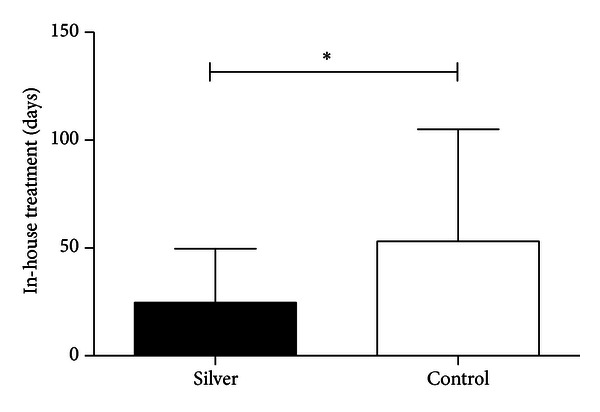
Clinical course of the group with implantation of a silver-coated megaprosthesis and the no silver group (mean value). Period of hospitalization in days. *P ≤ 0.05.

The duration of the stay in hospital averaged 36.5 days in the group with silver-coated megaprostheses after implantation. In the subgroup analysis, patients in the retrospective group with megaprosthesis without silver had a significantly longer stay in the hospital (MV: 72.1 days, *P* ≤ 0.001) (Figure 7). 

#### 3.4.3. Functional Outcome

Seven patients were able to move the operated upon extremity without using orthopedic aids (5 patients from the infection prophylaxis group) within 12 months after surgery. Nine patients (6 patients from the infection group) were able to walk more than 200 m using auxiliary supports (e.g., forearm crutches, rolling walker). As mentioned above, a reinfection occurred in one patient such that the thigh had to be amputated (infection prophylaxis group); in one patient, the inserted megaprosthesis became dislocated (infection group). For the dislocated megaprosthesis, a closed reposition was carried out, and the following outcome was uneventful. In all patients, the X-ray control in the two planes at day 5, after 6 weeks, after 3 months, and after 12 months showed a regular implant position without any signs of loosening or infection (except for the amputated patient). The wound healing was uneventful and without prolonged secretion, and the scar 12 months postoperatively showed no inflammatory signs.

#### 3.4.4. Survival

All 18 patients survived 12 months postoperatively. Indications of silver intoxication could not be proven clinically. Also, chemical laboratory analyses that were conducted during routine checks did not indicate any silver intoxication. Neither liver enzymes such as alanine aminotransferase (ALT) and aspartate aminotransferase (AST) nor renal serum parameters such as creatinine concentrations changed during the entire trial period, as a sign of silver-dependent organ damage, significant when compared to the control group.

## 4. Discussion

Due to an increasing infection rate with a rising resistance of bacteria against the usually applied antibiotics, the development of endoprosthetic metallic coatings that can perhaps lower infection rates is essential. Such prostheses would be particularly valuable to infection-prone patients with a malign underlying disease or to patients with a disturbed immune defense from pre-existing conditions, age or as a consequence of severe trauma. Thus, Gosheger et al., Ahrens et al., and Hardes and Von Eiff demonstrated that the silver coating of a prosthesis can decrease the reinfection rate in an animal experiment or an oncological patient cohort due to the release of silver ions [4, 9, 10]. Moreover, Hardes et al. illustrated the kinetic course of the silver ion concentration in the peripheral blood [3]. In the present study, the concentration of released silver ions in the direct surroundings of the prosthesis (wound fluid) was measured for the first time. This sample represents a common area for bacterial prosthesis infection in which systematically applied antibiotics work increasingly poorly. It is well known that bacteria, especially near the prosthesis, have become increasingly resistant to antibiotic eradication due to virulence factors, such as the formation of a biofilm [14, 15]. When comparing the measured silver concentrations with those values published in the recent literature, it is evident that the values measured in this study are apparently lower. For example, Straub et al. demonstrated bactericide effects of silver ions on gram-negative periodontal pathogens starting at values of 0.5 ppm, and Zhao and Stevens Jr. achieved bactericide effects of 2.0 ppm in an *in vitro* study [16, 17]. As mentioned before, it is unclear—based on the current literature—whether an average silver ion concentration of 0.08 ppm that has been measured in this study with megaprostheses provides sufficient levels of bactericide action. Nevertheless, it was possible to demonstrate clinical effects. The initial objective of this study was to clarify in a first step whether silver ions can be found in the direct surroundings of the prosthesis and if there are any insights with regard to the clinical course depending on measured concentrations. In the future, further studies shall demonstrate whether the concentrations measured in wound fluid that were collected in the immediate proximity of the prosthesis may have *in vivo* bactericide effects.

It must be taken into account that silver ions may build complexes with serum albumin [18]. Schierholz et al. showed in their study that this serum albumin silver complexing may reduce the bactericidal effects of silver [19]. Gosheger et al. suggest in their study that bactericidal effects may be reduced due to dilution, whenever body fluids get in contact with silver ions [9]. This is a basic relationship that is true for all applications of medicines. In our study, the amount of active silver ions appeared large enough, since a significant reduction of infection rates has been observed when compared to the retrospective group with megaprosthesis without silver.

Some evidence suggests that silver coatings may not have exclusively positive effects on patient outcome. Silver coatings have been implicated in osteolyses and postoperative prosthesis loosening, and the positive effects must be measured against the negative in each individual case [20, 21]. Rosengren and Dixon described in a review that silver-impregnated dressings have demonstrated no advantage in the healing of chronic wounds in a dermatological patient cohort [22]. Again, the decision to implant a megaprosthesis must be made on a case-by-case basis.

When considering the reinfection rate, there is a significant difference between endoprostheses with and without silver coatings. Although there is no statistically significant correlation between the increased concentration of silver ions in the wound fluids or in the peripheral blood and the reinfection rate, due to the small number of cases, the indication of a direct connection seems to be reasonable. As mentioned above, this conclusion is confirmed by the current literature [10]. Interestingly, in the traumatological patient cohort of patients who already had existing or previous infections, the reinfection rate decreased. This connection can currently not be exhaustively discussed due to the lack of the current literature regarding a traumatological patient cohort.

Due to the small group size and the concomitant low level of significance, it remains unclear whether the concentration of released silver ions in the immediate surroundings of the prosthesis has an influence on the clinical course. However, our results are promising. Considering the course of the CRP, patients with a relatively large amount of released silver ions show a faster decrease in the inflammatory marker CRP. The only case of reinfection in the described patient cohort shows that an insufficient quantity of loosened silver ions may be ineffective.

However, conclusions regarding a minimum concentration can currently not be made. To show a minimum concentration, a larger patient cohort or another animal experiment is needed. Moreover, the data show that patients which initially had a higher concentration in the systemic venous blood (7th day postoperatively) had a lower concentration of silver ions in the wound fluids. Thus, the too fast removal of the loosened silver ions and the resulting decrease from the effective concentration near the prosthesis seem to delay the decrease in inflammation. This connection also cannot be exhaustively discussed due to the current literature. In a recent *in vitro* study, however, Wu et al. described that the surrounding level of fluid and immersion time influence the release of silver ions. Unfortunately, this study does not aim to assess the effect of the released silver ions on bacterial infections [23].

In the present study, no toxic side effects from silver were found in the patients. This corresponds to previous studies by Gosheger et al. and Hardes et al., as well as other authors [3, 9, 24]. The minimum doses mentioned in the literature of approximately 4–6 g, for example, to cause argyria, are not approached in the present study [25–27]. However, as an accumulation of silver ions is in principle possible, Hardes et al. concluded that this must be considered at all times [3]. Thus, a case report by Sudman et al. describes a thousandfold increase of the silver serum level compared to baseline. The patient described in this study suffered from this complication 5 years after the installation of a hip endoprosthesis. This hip endoprosthesis was implanted with PMMA bone cement that had been supplied with 1% silver. This patient showed a peripheral neuropathy, but it remained unclear whether the supplied silver ions alone caused the complication [28]. Further case reports have discovered other potential complications, including a greasy degeneration of liver, heart, and kidneys [29, 30].

The significant reduction of the hospitalization period and the decrease in revision surgeries, especially in the patient group with previous infections, illustrate the potential significance of silver-coated megaprostheses. Here, we have analyzed the benefit of these prostheses for the first time in a traumatological patient cohort. It is apparent that silver-coated prostheses can be used not only for prophylaxis but also for the decrease of the reinfection risk. In the group of traumatological patients, no reinfections occurred; this result is particularly relevant because the average age in the traumatological group was 69.5 years. Such patients in particular should not undergo frequent surgery, as their operative risk increases with age. The same applies to patients with multiple injuries, whose nosocomial infection risks only increase with longer stays in the intensive care unit [31].

In the traumatological cohort in particular, using the silver-coated megaprosthesis often represented the final option before amputation to treat the infection or fight sepsis. In the present study, acute infections were often in transition towards chronic inflammations in the infection group, or the patients were in sepsis due to an osseous and soft tissue-driven source of infection. Therefore, it remains remarkable that all of the described patients retained the extremity concerned and were discharged from hospital as mobile, only using auxiliaries like forearm crutches. The danger of amputation as the last option to control or treat infection is extensively described in the literature [32–36].

Finally, in the light of dwindling resources, the economic factor has to be considered. The silver-coated megaprosthesis is admittedly 5–7% more expensive than the nonsilver-coated prosthesis [9]. However, the significant decrease in the period of hospitalization and the decrease in revision surgeries must be taken into account as relevant cost factors [37, 38].

*Limitations*

1. In this study, the group size was too small to prove highly significant results. Additional prospective studies with larger cohorts are required to achieve statistically reliable results.
2. A prospective randomized study regarding the use of silver-coated against nonsilver-coated endoprostheses could not be carried out due to ethical concerns. Especially in the traumatological group, the use of a silver-coated endoprosthesis was often the last possible option before amputation. From a scientific point of view, the realization of such a study would be required to increase the evidence level.
3. The period of postoperative evaluation was 12 months. In the context of the total joint registry data, this is an early followup. In particular, this study does not allow for a statement of the implant survivorship.
4. A histological examination in still inserted prostheses was not yet possible. The analysis of, for example, foreign body reactions at the cellular level was thus not possible. It is important to carry out further analyses in the future, for example, by removing prostheses or post mortem.

## 5. Conclusions

In the present study, increased silver concentrations were detected in the immediate surroundings of silver-coated prostheses. Our data suggest that silver release does improve clinical outcome. For the first time, the positive effect of silver-coated megaprostheses was demonstrated for trauma patients and tumor patients.

## Figures and Tables

**Table 1:** Demographic descriptions of the patient cohort, the indication for the silver prosthesis, an illustration of the joint concerned, and the total silver mass of the respective prosthesis.

Patient no.	Age at the time of surgery	Gender	Type of fracture/tumor	Indication silver	Type of prosthesis	Silver mass (g)
1	37	w	Secondary open distal femur fracture	Infection Plate osteosynthesis	Proximal Tibia, distale femur	0.76
2	67	m	Fractured acetabulum	Infection THA	Proximal femur	0.62
3	83	w	Medial fracture of the femur neck	Infection dual head prosthesis	Proximal femur	0.46
4	73	m	Periprosthetic fracture of the femur	Infection THA	Proximal femur	1.69
5	50	m	Medial fracture of the femur neck	Infection dual head prosthesis	Proximal femur	0.46
6	81	w	Medial fracture of the femur neck	Infection dual head prosthesis	Proximal femur	0.67
7	83	w	Subtrochanteric fracture of the femur	Infection Plate osteosynthesis	Proximal femur	0.52
8	89	w	Medial fracture of the femur neck	Infection dual head prosthesis	Proximal femur	0.46
9	63	w	Pertrochanteric fracture of the femur	Infection intramedullary nail	Proximal femur	0.62
10	45	w	Secondary open supracondylar humerus fracture	Infection elbow prosthesis	Distal humerus, prox. ulna	0.4
11	46	w	Osteosarcoma	Loosened TKA	Proximal tibia, distale femur	1.33
12	55	w	Metastasis renal cell carcinoma head of the humerus	Prophylaxis	Proximal humerus	0.42
13	64	w	Metastasis cervix carcinoma distal humerus	Prophylaxis	Distal humerus, prox. ulna	0.95
14	71	m	Metastasis adenoca prox. femur	Prophylaxis	Proximal femur	1.03
15	24	m	Ewing's sarcoma prox. tibia	Prophylaxis	Proximal tibia, distal femur	1.24
16	66	w	Chondrosarcoma humerus	Prophylaxis	Proximal humerus	0.97
17	24	m	Osteosarcoma femoral shaft	Prophylaxis	Total femur	0.68
18	60	w	Metastasis mamma ca prox. femur	Prophylaxis	Proximal femur	0.75

Patients 1–10: infection group; patients 11–18: infection prophylaxis group; g: gram; THA: total hip arthroplasty; TKA: total knee arthroplasty.

**Table 2:** Infection group: clinical course prior to the implantation of a silver-coated megaprosthesis.

Patient no.	Days until clinically definite infection	Pathogen of the infection	Revision operations	Days of hospitalization prior to SMP implantation	Days after SMP implantation
1	36	Staph. epi.	7	66	31
2	18	Enterobacter cloacae, Staph. epi.	5	48	31
3	15	Staph. epi.	12	73	44
4	40	Staph. epi, Enterococcus faec., Corynebacterium	7	227	92
5	18	Staph. epi, Enterococcus faec.	5	74	24
6	13	Staph. epi.	4	47	27
7	8	Enterobacter cloacae	5	60	34
8	14	Not demonstrable	2	7	54
9	12	Enterococcus faec.	7	105	23
10	9	Not demonstrable	2	14	5

Staph: *Staphylococcus*; epi: *epidermidis*; feac: faecalis; SMP: silver-coated megaprosthesis.

**Table 3:** Demographic descriptions of the retrospective group, an illustration of the joint concerned, and the infection rate.

Patient no.	Age at the time of surgery	Gender	Type of fracture/tumor	Type of prosthesis	Infection yes/no
1	71	m	Periprosthetic fracture of the femur	Proximal femur	yes
2	56	m	Subtrochanteric fracture of the femur	Proximal femur	no
3	83	w	Pertrochanteric fracture of the femur	Proximal femur	yes
4	84	w	Medial fracture of the femur neck	Proximal femur	yes
5	52	m	Medial fracture of the femur neck	Proximal femur	yes
6	85	w	Pertrochanteric fracture of the femur	Proximal femur	no
7	96	w	Medial fracture of the femur neck	Proximal femur	no
8	90	w	Medial fracture of the femur neck	Proximal femur	yes
9	88	w	Pertrochanteric fracture of the femur	Proximal femur	no
10	83	w	Pertrochanteric fracture of the femur	Proximal femur	no
11	85	m	Medial fracture of the femur neck	Proximal femur	no
12	78	w	Pertrochanteric fracture of the femur	Proximal femur	yes
13	85	m	Periprosthetic fracture of the femur	Proximal femur	no
14	82	w	Periprosthetic fracture of the femur	Proximal femur	no
15	88	w	Medial fracture of the femur neck	Proximal femur	yes
16	56	w	Metastasis mamma carcinoma prox. femur	Proximal femur	no
17	54	m	Metastasis oropharyngeal Ca femoral shaft	Proximal femur	no
18	60	w	Metastasis mamma ca prox. femur	Proximal femur	no
19	78	m	Metastasis cancer of unknown primary	Proximal femur	no
20	55	w	Metastasis mamma ca prox. femur	Proximal femur	no
21	70	w	Metastasis mamma ca prox. femur	Proximal femur	no
22	31	m	Metastasis chondrosarcoma femoral shaft	Proximal femur	no
23	57	w	Metastasis hepatocellular carcinoma femur	Proximal femur	no
24	68	m	Metastasis Hypernephroma femur proximal	Proximal femur	no
25	75	m	Plasmacytoma femur proximal	Proximal femur	no
26	76	w	Metastasis corpus uteri Prox. femur	Proximal femur	no
27	75	m	Plasmacytoma femur proximal	Proximal femur	no
28	73	m	Metastasis kidney ca prox. femur	Proximal femur	no
29	75	m	Metastasis chondrosarcoma femoral shaft	Proximal femur	no
30	75	w	Metastasis kidney ca prox. femur	Proximal femur	no
31	76	w	Metastasis rectum ca prox. femur	Proximal femur	no

Patients 1–15: trauma group; patients 16–31: tumor group.
